# *Helicoverpa armigera* preference and performance on three cultivars of short‐duration pigeonpea (*Cajanus cajan*): the importance of whole plant assays

**DOI:** 10.1002/ps.7230

**Published:** 2022-10-27

**Authors:** Trevor M. Volp, Myron P. Zalucki, Michael J. Furlong

**Affiliations:** ^1^ School of Biological Sciences The University of Queensland St Lucia Australia; ^2^ Department of Agriculture and Fisheries Agri‐Science Queensland Toowoomba Australia

**Keywords:** host–plant resistance, oviposition behaviour, larval performance, bioassay, antibiosis, antixenosis

## Abstract

**BACKGROUND:**

*Helicoverpa armigera* is a major pest of pigeonpea (*Cajanus cajan*). Efforts to develop pigeonpea varieties resistant to *H. armigera* attack have been met with limited success, despite reports of high levels of resistance to *H. armigera* in wild relatives of pigeonpea and reports of low to moderate levels of resistance in cultivated varieties. Here we examined *H. armigera* oviposition preference and larval performance on whole plants of three cultivars of short‐duration pigeonpea: a susceptible control (ICPL 87) and two cultivars with purported host–plant resistance (ICPL 86012 and ICPL 88039).

**RESULTS:**

In our no‐choice oviposition experiment, *H. armigera* laid similar numbers of eggs on all three cultivars tested, but under choice conditions moths laid slightly more eggs on ICPL 88039. Larval growth and development were affected by cultivar, and larvae grew to the largest size (weight) and developed fastest on ICPL 86012. Moths laid most of their eggs on floral structures, sites where subsequent early instar larvae overwhelmingly fed. Experimentally placing neonate larvae at different locations on plants demonstrated that larvae placed on flowers experienced greater survival, faster development, and greater weight gain than those placed on leaves. The type and density of trichomes (a potential resistance trait) differed among cultivars and plant structures, but larvae selected to feed at sites where trichomes were absent.

**CONCLUSION:**

Future work examining host–plant resistance against *H. armigera* should incorporate the behavioural preference of moths and larvae in experiments using whole plants as opposed to bioassays of excised plant parts in Petri dishes. © 2022 The Authors. *Pest Management Science* published by John Wiley & Sons Ltd on behalf of Society of Chemical Industry.

## Graphical abstract

We examined *Helicoverpa armigera* preference and performance on three pigeonpea (*Cajanus cajan*) cultivars. Our findings emphasize the importance of using whole plants if host–plant resistance evaluations are to be meaningful.

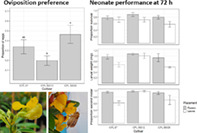

## 1 INTRODUCTION

Host–plant resistance can be a critical component of strategies to manage insect pests of agricultural crops. Modern agricultural pest management typically relies on synthetic insecticides but developing insect resistant and/or tolerant crop cultivars can decrease reliance on these compounds.^1^, ^2^ Host–plant resistance, through both conventional and transgenic approaches, has experienced many successes.^1^, ^3^ Yet limited progress in the development and adoption of these technologies has been made in several staple crops.^4^ There are several limitations to progressing development of insect resistant crops, including overreliance on ‘Petri dish assays’ of detached plant parts in the laboratory, which are often misleading when it comes to interpreting insect responses to plant traits.^5^, ^6^, ^7^

*Helicoverpa armigera* (Hübner) (Noctuidae) is a key pest of agriculture that attacks many crops throughout Asia, Europe, Africa, Australia, and South America.^8^, ^9^, ^10^, ^11^ Due to the pest's propensity to rapidly evolve resistance to insecticides,^12^, ^13^, ^14^ there is an emphasis on developing sustainable, less chemically intense management practices. Integrated pest management tactics that are already used for managing *H. armigera* include biopesticides,^15^ biological control,^16^, ^17^ and transgenic crops.^13^ Conventional host–plant resistance, however, remains underutilized in *H. armigera* management.^18^

*Helicoverpa armigera* is arguably the major biotic constraint to the global production of pigeonpea (*Cajanus cajan*, (L.) Millsp.), a major legume crop with an average annual yield of ~5 million tonnes.^19^ *Helicoverpa armigera* infestations in pigeonpea cause substantial yield loss due to larvae feeding on crops during their susceptible reproductive stages.^20^, ^21^ In India, where the majority of global pigeonpea production occurs,^19^ subsistence farmers rely on synthetic insecticides to control the pest.^22^ Traditionally farmers have grown ‘long’ duration pigeonpea cultivars (> 250 days to maturity), that may tolerate or avoid *H. armigera* attack by flowering and podding during cooler periods when pest pressure is lower.^20^, ^23^ However, modern pigeonpea breeding has shifted towards ‘short’ duration cultivars (< 140 days to maturity). These cultivars appear to be at even greater risk of yield loss due to *H. armigera*, purportedly due to their compact growth, determinate flowering type, and clustered racemes making these cultivars either more attractive to pests, more suitable for larval establishment, or less tolerant of pest injury.^20^, ^21^, ^24^ Incorporating plant defence traits into commercial pigeonpea varieties provides an opportunity to reduce the current dependency and unsustainable use of synthetic insecticides in pigeonpea production.

A substantial body of research has aimed to develop pigeonpea cultivars with plant defence traits to manage *H. armigera*.^18^, ^25^ These traits can confer resistance (traits that reduce the level of injury caused by the pest either by reduced oviposition or poor performance of immatures) or tolerance (traits that allow the plant to recover from injury).^26^ Thousands of pigeonpea accessions have been screened for resistance and tolerance to *H. armigera*.^21^, ^27^ No genotypes exhibit complete defence against the pest, but many demonstrate degrees of resistance and/or tolerance. High levels of resistance have been recorded in uncultivated wild relatives of pigeonpea,^28^ purportedly due to non‐glandular trichomes (on calyxes and pods) and polyphenols (in leaves and pods).^29^, ^30^, ^31^ In cultivated pigeonpea, resistance has been identified in several agronomically‐suitable genotypes in the form of ovipositional non‐preference and larval antibiosis.^32^, ^33^, ^34^, ^35^

Historically, host–plant resistance screening in pigeonpea has relied on small cage oviposition experiments using excised inflorescences^28^, ^33^, ^35^ and laboratory bioassays examining larval antibiosis on detached plant parts.^28^, ^31^, ^32^, ^34^, ^36^ Despite the limitations of such approaches, even recent studies persist in screening pigeonpea genotypes with detached‐leaf assays.^37^, ^38^, ^39^, ^40^ The conditions created in these experiments ignore the behavioural ‘choice’ of *H. armigera* larvae, in particular intra‐plant movement as larvae tend to move up plants and select floral structures or terminal leaves as feeding sites,^41^, ^42^, ^43^ along with detecting and avoiding induced plant defences.^6^, ^44^

In this study we evaluated three cultivars of short‐duration pigeonpea for resistance against *H. armigera* (ICPL 87, ICPL 86012, and ICPL 88039) (Table 1). ICPL 87 is regarded as a highly *H. armigera*‐susceptible cultivar of pigeonpea and is regularly used in studies as a susceptible check.^36^ Whereas, both ICPL 86012 and ICPL 88039 have been reported to have low to moderate levels of resistance in the form of ovipositional non‐preference and larval antibiosis.^32^, ^33^, ^34^, ^35^ Here we: (i) examine *H. armigera* ovipositional preference among pigeonpea cultivars using whole plants, (ii) detail where *H. armigera* moths lay their eggs on pigeonpea plants, and (iii) experimentally measure how early instar performance (survival, growth, and development) relates to ovipositional preference, again on whole plants.

**Table 1:** Pigeonpea cultivars used in this study and previously published results from host‐plant resistance screening experiments

Cultivar	Determinacy	Flower colour	Previous resistance screening findings	Reference(s)	Methodology used
ICPL 87	Determinant	Yellow	Highly preferred for oviposition, larval feeding, and larval development. Used as a susceptible check.	^31^, ^32^, ^33^, ^34^, ^35^, ^38^	Various – including field experiments, caged whole plant assays, and excised plant part laboratory assays. Most studies have relied upon detached plant parts (particularly leaves) in the laboratory.
ICPL 86012	Determinant	Red/yellow	Ovipositional antixenosis – fewer eggs laid under choice and no‐choice conditions.	^33^	Choice – outdoor cages, whole plants No‐choice – laboratory cages, excised inflorescences.
			Larval antibiosis – lower larval and pupal weights, and slower development.	^32^	Excised plant structures in Petri dishes.
ICPL 88039	Indeterminate	Yellow	Oviposition antixenosis – moths laid fewer eggs under no‐choice and multi‐choice conditions.	^35^	No‐choice – laboratory cages, excised inflorescences Multi‐choice – laboratory cages, excised inflorescences.
			Larval antibiosis – larvae developed slower on ICPL 88039 leaves compared to ICPL 87 leaves.	^34^	Excised plant structures in 250 mL plastic cups in addition to leaf and pod material incorporated into artificial diet.

## 2 MATERIALS AND METHODS

### 2.1 Plants

Pigeonpea plants of the three selected cultivars (Table 1) were grown in a controlled temperature glasshouse (27 ± 4 °C day, 25 ± 4 °C night), under natural photoperiod, located at the Queensland Government Department of Agriculture and Fisheries facility in Toowoomba, Australia (−27.534137, 151.929201). Pigeonpea seeds were sourced from the pigeonpea germplasm collection of the Queensland Department of Agriculture and Fisheries, originally derived from the Australian Grains Genebank. Seeds were placed on filter paper moistened with distilled water in plastic Petri dishes (90 mm diameter) and held in an incubator at 25 ± 1 °C under darkness. After emergence of the radicle, germinating seeds were transferred to 4 L pots filled with a 2:1 mix of Searles Premium™ potting mix and sand. Plants were watered regularly, as required, and no additional fertilizer was provided. The cultivars differed in their time to flowering so we used weekly plantings to ensure all cultivars could be compared at the flowering stage. Plants used in experiments were regularly checked for the presence of any glasshouse pests (mites, thrips, and whitefly) and these were physically removed if present. No insecticides were applied to plants.

### 2.2 Insects

A laboratory colony of *H. armigera* was established from collections of moths and larvae from various field crops of southeast Queensland, Australia. Adult moths were kept in 5‐L plastic buckets and supplied with 10% sucrose solution using a cotton wick in 70 mL plastic specimen jars. A hole was cut in the bucket lid and the edges of the lid were used to secure nappy liner (bamboo rayon) which was used as an oviposition substrate. Eggs were removed daily, washed in 1% sodium hypochlorite solution, rinsed with distilled water, and collected onto filter paper (Whatman™) using vacuum filtration. Filter paper was air dried, then placed in Petri dishes (90 mm diameter) which were sealed with parafilm, until neonates hatched. Upon hatching, neonate larvae were immediately placed in plastic containers (17.5 cm × 12 cm × 6 cm) and provided with soybean flour‐based artificial diet – recipe modified from Teakle and Jensen^45^ (Supporting Information Table S1). When they developed to the third instar, larvae were transferred into 32‐well plastic trays – containing fresh diet – where they remained until pupation. Pupae were washed in 1% sodium hypochlorite and placed on paper towel in plastic containers (17.5 cm × 12 cm × 6 cm) within mesh emergence cages (60 cm × 60 cm × 60 cm, polyester mesh, Bugdorm™) until moth emergence. Moths and larvae were kept in a temperature‐controlled room [25° ± 2 °C; 12 h:12 h, light/dark artificial light; 60% relative humidity (RH)] with access to an external window for natural light.

Moths for oviposition experiments were obtained from the laboratory colony. Pupae were separated by sex and placed in separate emergence cages. Pupae were checked daily, and upon eclosion moths were removed and placed in mating cages (5 L buckets); 20 moths (*n* = 10 females, *n* = 10 males) per cage. Moths were allowed three nights to mate and to commence laying eggs. After three nights, females were individually removed and placed into oviposition cages (68.5 cm × 68.5 cm × 122 cm, polyester mesh, Nasco™) in a glasshouse.

### 2.3 Oviposition choice experiment

**Figure 1:**
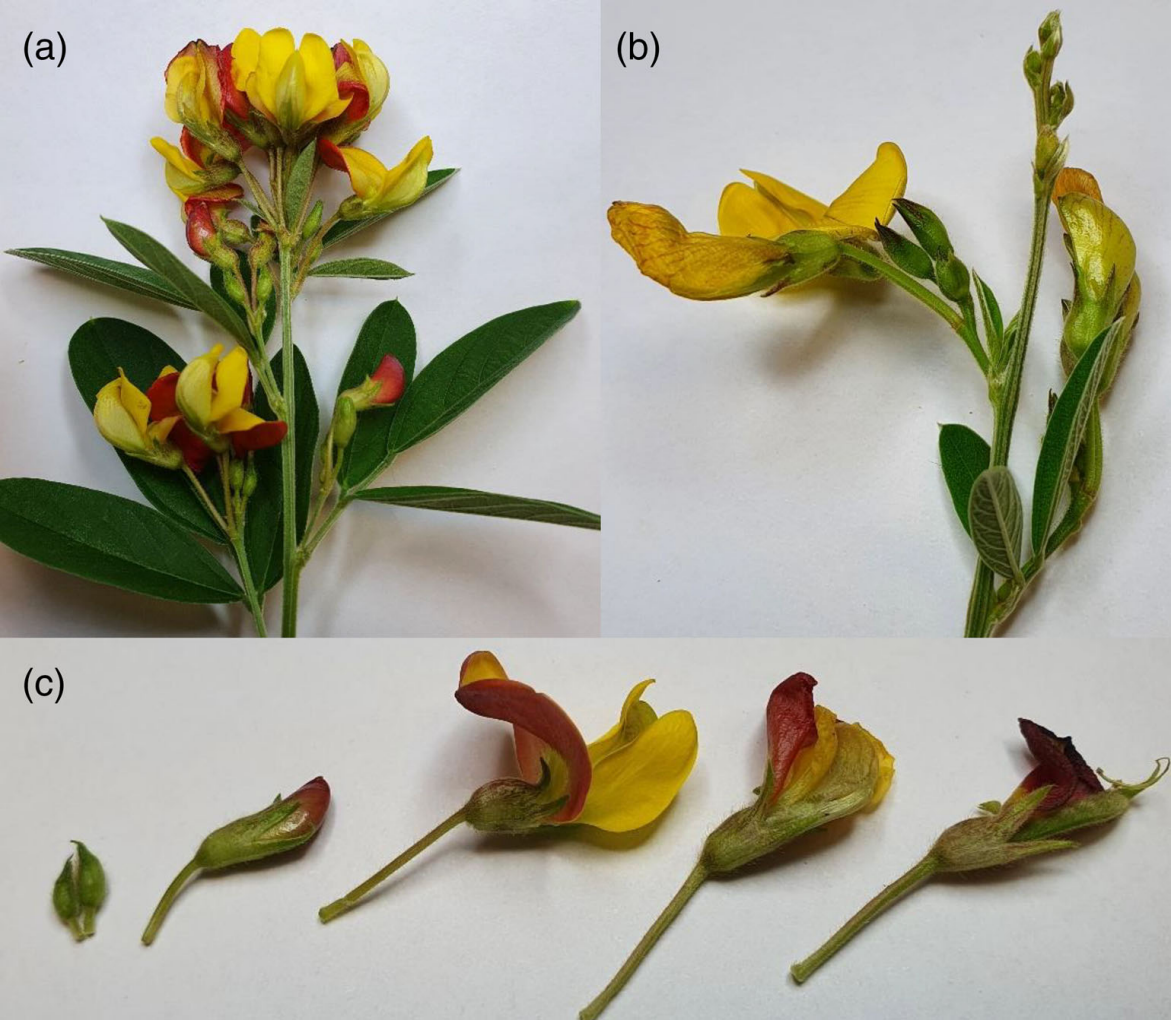
Pigeonpea reproductive structures. (a) The terminal raceme of the determinant cultivar ICPL 86012. (b) Indeterminate cultivar ICPL 88039 and the acropetal flowering pattern that results in a series of bud initials at growing tips. (c) Floral structures of ICPL 86012, from left to right: bud initials, bud, flower, spent flower, and a small pod.

For the oviposition choice experiment, oviposition cages contained one flowering plant of each cultivar (ICPL 87, ICPL 86012, and ICPL 88039). Plants were randomly allocated to a position in an equilateral triangle within the cage, potting mix‐filled pots were placed underneath shorter plants to ensure all plants were the same height from the base of the cage to avoid any influence of plant height on oviposition behaviour.^46^ Single females were placed in cages at approximately 08:00 h and were left in the cage to lay eggs overnight. If a female did not lay eggs on her first night, she was kept in the cage for another 24 h and plants were checked for eggs the next morning. The number and location of all eggs laid were recorded, plant height was measured, and the number of different structures [expanded trifoliate leaves, small (expanding) or large (seed‐filling) pods and floral structures] were counted on each plant. Floral structures were separated into bud initials (petals were yet to grow out of the calyx), buds (petals had grown out of the calyx, but were yet to open), flowers (opened petals), and spent flowers (petals had opened, the flower had been fertilized, and petals had commenced desiccation) (Fig. 1). Plants were retained and monitored for egg hatching to ensure females were laying fertile eggs.

### 2.4 Oviposition no‐choice experiment

For no‐choice oviposition assays, mated female moths were obtained in the same manner as those in the choice experiment. Individual female moths were placed singly in an oviposition cage in the glasshouse with a single flowering plant of one of the three test cultivars. As in the choice experiment, plants were examined for eggs the next morning. If females did not lay, they were left with the plants for a further 24 h to oviposit. We recorded the number of eggs laid and their location, and measured plant traits as in the first experiment.

### 2.5 Larval performance

The performance of early instar *H. armigera* larvae was assessed on flowering plants of the three pigeonpea cultivars in the glasshouse. *Helicoverpa armigera* eggs from the laboratory colony were closely monitored upon reaching the ‘blackhead’ stage (i.e. just prior to hatching). Neonate larvae were collected as soon as they had hatched and finished feeding on their egg chorion, larval hatching was synchronous among egg batches, and neonates typically consumed their egg chorion within < 10 min of hatching (at 27 °C in the glasshouse). To examine the effect of oviposition site on larval performance, neonates were either placed on flowers or leaves on one of the three cultivars at flowering.

On each plant, ten neonates were placed on the top two nodes with the appropriate plant structures (five larvae per node). Larvae were left to feed on plants for 72 h, after which plants were destructively harvested and searched carefully for larvae. This process involved opening every reproductive structure on plants and recording the location of each larva that was found, whether it was alive or dead, and its stage of development. To determine the average weight of surviving larvae, live larvae were weighed on an analytical balance (HR‐250AZ; A&D, delaide, South Australia) to the nearest 0.1 mg. The experiment consisted of a factorial design with three levels for cultivar and two levels for neonate placement location, with five blocked replicates.

### 2.6 Quantifying trichomes

We quantified the densities of different trichomes on the separate plant structures of the three pigeonpea cultivars. Using glasshouse grown flowering plants, we examined the locations where most oviposition and larval feeding occurred (flower calyxes, flower petals, and adaxial and abaxial leaf surfaces) under a stereo microscope (SMZ800N; Nikon, Tokyo, Japan). Using an ocular grid, we quantified the number of different trichome types as described in Romeis *et al*.^30^ Trichomes on pigeonpea can be separated into five types (A–E). Type A trichomes are tall (~557 μm) and glandular, whereas, Type B are short (86 μm), globular and glandular. Both Type C and Type D trichomes are non‐glandular, Type C are short (~116 μm) whereas Type D are long (~502 μm). Finally, Type E trichomes are multi‐lobed, very short (< 50 μm) and glandular. Due to their typical very low density, we did not record Type E trichomes in this study. Trichomes were quantified under 8× magnification, using a grid size of 1.56 mm^2^ for Types A, B, and D trichomes, and Type C trichomes were quantified using a grid area of 0.156 mm^2^ due to their very high density. We used flowers and leaves from the first fully expanded node for trichome type and density measurements. We made three observations for each plant structure per plant and we repeated this for ten plants of each cultivar.

### 2.7 Statistical analysis

Egg count data from the oviposition choice experiment were converted to proportions (the number of eggs per cultivar divided by total number of eggs laid in each cage) prior to analysis, due to the large variation in the number of eggs laid by *H. armigera* moths. Residual plots indicated data transformation was not necessary for the proportional data and one‐way analysis of variance (ANOVA) was used to compare the proportion of eggs laid on different cultivars in the choice experiment.

For the no‐choice experiment, egg count data were analysed with a Kruskal–Wallis test, as the data did not meet the assumption of normality for one‐way ANOVA, even after transformation. We used one‐way ANOVAs to compare the plant morphometric data (plant height, mainstem node count, and counts of plant structures) collected during the oviposition experiments. We analysed plant morphometric data from the choice and no‐choice experiments separately.

We examined the within‐plant distribution of *H. armigera* eggs in the oviposition no‐choice experiment to determine if the distributions of eggs differed among cultivars. We used one‐way ANOVAs to analyse the distribution of eggs on different plant structures (leaves *versus* floral structures) by calculating the proportion of eggs laid on either leaves or floral structures out of the total number of eggs laid per plant (replicate), which we then analysed using one‐way ANOVAs with cultivar as the fixed effect. We also examined the proportion of eggs laid on the different floral structures (bud initials, buds, flowers, and spent flowers) by taking the number of eggs laid on a single type of floral structure and dividing this by the total number of eggs laid per plant, then comparing that proportion among cultivars. When analysing the within‐plant distribution of eggs in the oviposition no‐choice experiment we removed plants on which < 10 eggs had been laid to avoid inflated proportional values; in total five replicates were removed, three belonging to ICPL 88039 and two belonging to ICPL 86012.

We also analysed the distribution of eggs within plant structures (i.e. among different sublocations). To do this we pooled data from all three cultivars and analysed the proportion of eggs laid in a different sublocations for different plant structures. We classified sublocations as the abaxial or adaxial surfaces of leaves, and pedicels, calyxes, and petals for floral structures (except for bud initials which do not have petals present). We used replicate (i.e. a cage with a single plant and single moth) as a blocking factor to control for non‐independence and also removed replicates where a plant structure received fewer than ten eggs to avoid over inflated proportional values.

For the larval performance experiment, we performed two‐way ANOVAs to examine response variables (final larval location, survival, weight, and larval development) with cultivar and larval placement location used as fixed effects, and replicate as a blocking factor. For comparing trichome densities at a specific location (e.g. calyx, abaxial leaf surface, or adaxial leaf surface), we calculated the mean of the three observations per plant structure per plant and then used one‐way ANOVAs with cultivar as the fixed effect. All statistical analyses were performed in R version 3.6.2,^47^ all graphs were created using the R package ‘ggplot2’,^48^ and when we found significant effects from ANOVA, *post hoc* multiple comparisons were performed using the Fisher's least significant difference (LSD) test in the R package ‘agricolae’.^49^

## 3 RESULTS

### 3.1 Oviposition experiments

**Figure 2:**
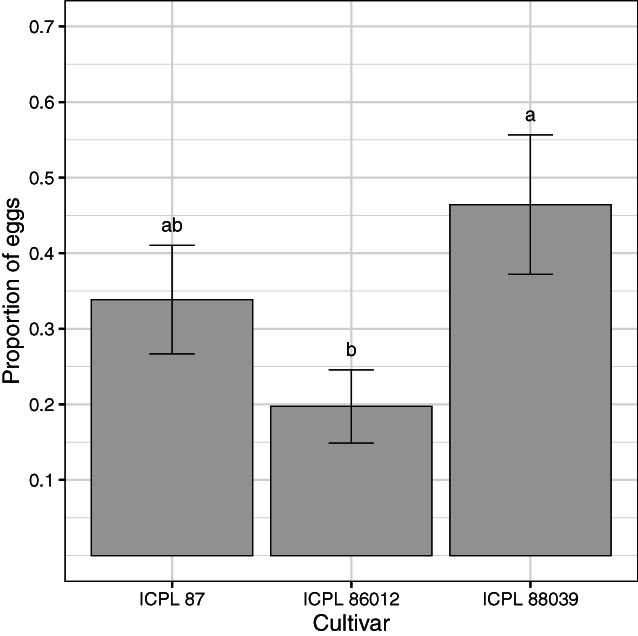
The mean proportion of eggs allocated to the three pigeonpea cultivars in the oviposition choice experiment (*n* = 17 replicates). Error bars are standard error of the means, and different letters above bars indicate a significant difference (*P* < 0.05) among cultivars according to Fisher's protected LSD test.

In the oviposition choice experiment, cultivar influenced oviposition choice as more eggs were laid on ICPL 88039 than the other two cultivars (*F*_2,48_ = 3.35, *P* = 0.044) (Fig. 2). The number of eggs laid by individual female moths in the choice experiment ranged between 35 and 477 in a single night. In the no‐choice experiment, there was no difference in the total number of eggs laid on the three cultivars of pigeonpea (*χ* ^2^_2_ = 4.52, *P* = 0.10) (Supporting Information Fig. S1). Out of the 90 single‐female cages run for the no‐choice experiment, only 34 moths laid eggs (Table S2). From the moths that laid eggs, 22 (65%) laid eggs on the first night after they were placed in the cage, the remainder laid on the second night. As in the choice experiment, there was large variability in the total number of eggs laid per female, ranging from a single egg to 643 eggs laid by an individual moth in a single night.

On average ICPL 88039 plants were taller, had more mainstem nodes, and more bud initials than the two determinate cultivars (Table 2). In the choice experiment cultivars differed in their number of trifoliate leaves (*F*_2,48_ = 14.99, *P* < 0.001), bud initials (*F*_2,48_ = 49.02, *P* < 0.001), the number of mainstem nodes (*F*_2,48_ = 82.36, *P* < 0.001) and their height (*F*_2,48_ = 140.5, *P* < 0.001). However, there was no difference among cultivars in the number of buds (*F*_2,48_ = 1.89, *P* = 0.16), flowers (*F*_2,48_ = 1.89, *P* = 0.16), nor spent flowers (*F*_2,48_ = 0.69, *P* = 0.51). In the no‐choice oviposition experiment, cultivars differed in their number of trifoliate leaves (*F*_2,31_ = 8.03, *P* = 0.016), bud initials (*F*_2,31_ = 24.37, *P* < 0.001), flowers (*F*_2,31_ = 12.08, *P* < 0.001), spent flowers (*F*_2,31_ = 3.87, *P* = 0.0316), mainstem nodes (*F*_2,31_ = 200.7, *P* < 0.001), and height (*F*_2,31_ = 236.2, *P* < 0.001) but there was no difference among cultivars for the number of buds (*F*_2,31_ = 2.35, *P* = 0.11). Small pods were only present on five out of 51 plants in the choice experiment and four of the 34 plants in the no‐choice experiment.

**Table 2:** Morphometric data (means ± standard errors) for plants used in oviposition experiments

Oviposition experiment	Cultivar	*N*	Plant height (mm)	Mean count (± standard error)					
				Nodes	Trifoliate leaves	Bud initials	Buds	Flowers	Spent flowers
	ICPL 87	17	419 ± 19 b	14.4 ± 0.7 b	22.9 ± 1.3 b	76.9 ± 5.9 b	26.2 ± 3.0 a	16.2 ± 1.5 a	17.2 ± 3.3 a
Choice experiment	ICPL 86012	17	406 ± 16 b	16.0 ± 0.5 b	28.9 ± 1.0 a	73.1 ± 5.0 b	31.6 ± 3.0 a	20.6 ± 1.6 a	22.6 ± 3.1 a
	ICPL 88039	17	786 ± 19 a	25.4 ± 0.7 a	33.5 ± 1.7 a	184.4 ± 13.6 a	35.5 ± 4.1 a	17.4 ± 2.1 a	19.8 ± 3.4 a
	ICPL 87	14	361 ± 12 b	13.7 ± 0.4 c	25.4 ± 2.0 b	83.6 ± 8.2 b	29 ± 3.5 a	19.4 ± 2.3 b	12.9 ± 1.9 ab
No‐choice experiment	ICPL 86012	12	372 ± 7 b	16.0 ± 0.2 b	35.1 ± 1.8 a	72.3 ± 4.5 b	37.6 ± 3.4 a	28.7 ± 2.4 a	15.9 ± 2.6 a
	ICPL 88039	8	675 ± 9 a	24.0 ± 0.4 a	30 ± 1.0 ab	158.0 ± 13.6 a	26.8 ± 4.1 a	11.1 ± 2.2 b	5.8 ± 3.0 b

Within an experiment, means within a column marked with different letters are significantly different (*P* < 0.05) according to Fisher's protected least significant difference (LSD) test. Results from analysis of variance are presented in the text.

**Figure 3:**
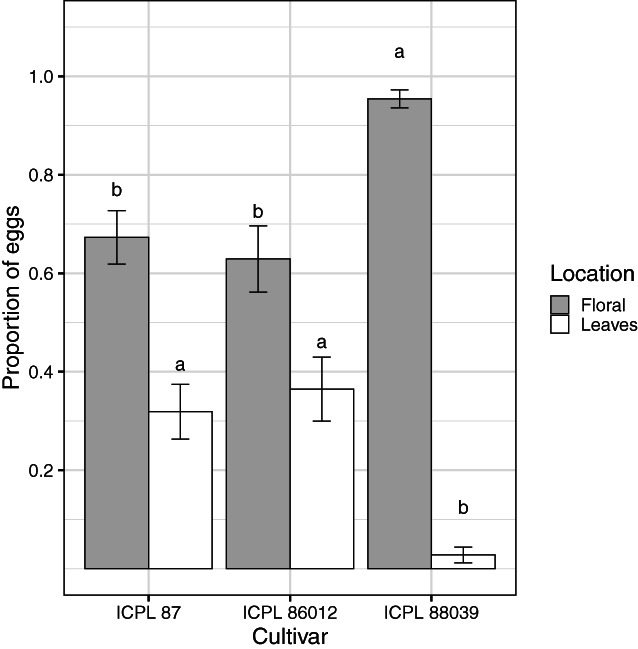
Proportion of eggs allocated to different plant structures in the oviposition no‐choice experiment. Grey bars indicate eggs laid on floral structures, whereas white bars indicate eggs laid on leaves. Floral structures include bud initials, buds, flowers, and spent flowers. Bars are the means of proportions and error bars are standard errors. Letters above bars indicates a significant difference (*P* < 0.05) among cultivars in the proportion of eggs allocated to a location as determined by Fisher's protected LSD test.

**Figure 4:**
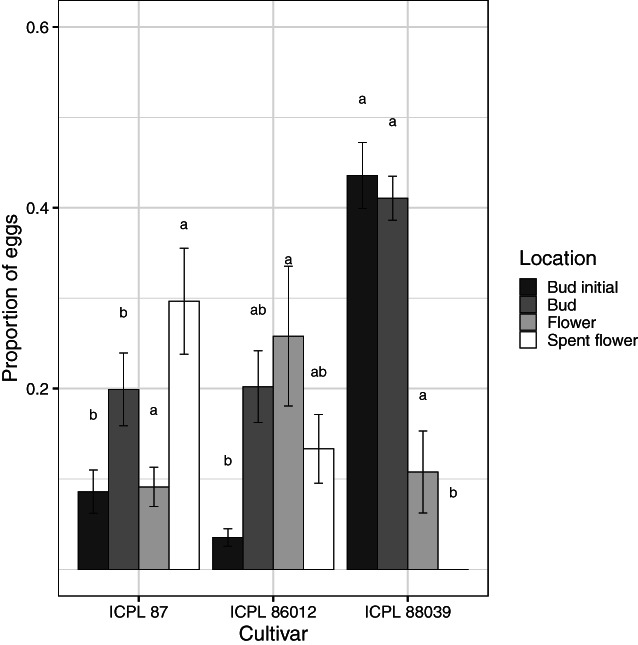
Proportion of eggs distributed on different floral plant structures in the oviposition no‐choice experiment. Bars are the means of proportions and error bars are standard errors. Proportion values are calculated as the number of eggs allocated on that structure divided by the total number of eggs laid per plant. Letters above bars indicates a significant difference (*P* < 0.05), determined by Fisher's protected LSD test, among cultivars in the proportion of eggs allocated to that specific floral structure.

In the no‐choice experiment, most *H. armigera* eggs were laid on floral structures (Fig. 3). The proportion of eggs on floral structures differed among cultivars (*F*_2,26_ = 5.20, *P* = 0.013), more eggs were allocated to floral structures when moths were provided with ICPL 88039 plants (Fig. 3). Conversely, the proportion of eggs allocated to leaves also differed among the cultivars (*F*_2,26_ = 5.61, *P* = 0.009), with more eggs laid on leaves on the two determinate cultivars (ICPL 87 and ICPL 86012). Pigeonpea cultivar influenced the amount of oviposition (expressed as a proportion of total eggs) on bud initials (*F*_2,26_ = 54.44, *P* < 0.0001), buds (*F*_2,26_ = 5.30, *P* = 0.012), flowers (*F*_2,26_ = 3.40, *P* = 0.049), and spent flowers (*F*_2,26_ = 6.4, *P* = 0.005). ICPL 88039 plants had proportionally more eggs on bud initials and buds, whereas eggs were more evenly distributed for ICPL 87 and ICPL 86012 (Fig. 4).

In the no‐choice experiment, eggs were non‐randomly distributed among different specific sublocations within plant structures (Tables 3 and 4). The distribution of eggs among sublocations differed for bud initials (*F*_1,13_ = 519.8, *P* < 0.001), buds (*F*_1,32_ = 46.58, *P* < 0.001), flowers (*F*_2_,_30_ = 9.16, *P* < 0.001), and spent flowers (*F*_2,28_ = 14.476.4, *P* < 0.001). On bud initials and buds, most eggs were laid on calyxes (Table 3), but on flowers and spent flowers the distribution of eggs did not differ between calyxes and petals. On all floral structures the fewest eggs were laid on pedicels. When eggs were laid on pigeonpea leaves, more eggs were laid on the abaxial surface (86%) than the adaxial surface (14%) (*F*_1,19_ = 69.01, *P* < 0.001) (Table 4). Of the 6267 eggs counted in the no‐choice experiment, only 32 were laid on pigeonpea stems (0.5%), 21 were laid on small pods (0.3%), and four were laid on leaf petioles (< 0.1%). These eggs are not presented in tables and were excluded from the analyses.

**Table 3:** Proportion of eggs at the identified sublocations out of all eggs laid on floral plant structures

Plant structure	Cultivar	*N*	Mean proportion (± standard error) of eggs at each structure sublocation		
			Calyx	Petals	Pedicel
Bud initials	ICPL 87	7	0.96 ± 0.04	NA	0.04 ± 0.04
	ICPL 86012	3	1	NA	0
	ICPL 88039	4	1	NA	0
	Total	14	0.98 ± 0.02 a	NA	0.02 ± 0.02 b
Buds	ICPL 87	8	0.61 ± 0.08	0.36 ± 0.07	0.03 ± 0.01
	ICPL 86012	5	0.80 ± 0.09	0.17 ± 0.08	0.03 ± 0.02
	ICPL 88039	4	0.79 ± 0.03	0.19 ± 0.04	0.02 ± 0.02
	Total	17	0.71 ± 0.05 a	0.27 ± 0.05 b	0.03 ± 0.01 c
Flowers	ICPL 87	8	0.30 ± 0.07	0.60 ± 0.10	0.10 ± 0.06
	ICPL 86012	5	0.45 ± 0.12	0.50 ± 0.13	0.05 ± 0.02
	ICPL 88039	3	0.80 ± 0.11	0.13 ± 0.07	0.06 ± 0.05
	Total	16	0.42 ± 0.07 a	0.51 ± 0.07 a	0.08 ± 0.03 b
Spent flowers	ICPL 87	11	0.29 ± 0.09	0.70 ± 0.09	0.01 ± 0.01
	ICPL 86012	4	0.52 ± 0.19	0.47 ± 0.18	0.01 ± 0.01
	ICPL 88039	0	NA	NA	NA
	Total	15	0.35 ± 0.08 a	0.64 ± 0.08 a	0.01 ± 0.01 b

Values presented are mean ± standard errors. Bud initials do not have any petals present, as they are yet to expand out of the calyx. Data for separate cultivars were combined due to small sample sizes (*N*), which varies for the different plant structure as some plants obtained no eggs on that structure and were therefore excluded from the analyses. Means marked with different letters are significantly different (*P* < 0.05) according to Fisher's protected least significant difference (LSD) test.

**Table 4:** Proportion of eggs on different sublocations locations within pigeonpea leaves

Plant structure	Cultivar	*N*	Mean proportion (± standard error) of eggs at each structure sublocation	
			Adaxial surface	Abaxial surface
Leaves	ICPL 87	12	0.18 ± 0.07	0.81 ± 0.07
	ICPL 86012	6	0.08 ± 0.04	0.91 ± 0.04
	ICPL 88039	2	0	1
	Total	20	0.14 ± 0.04 b	0.86 ± 0.04 a

Values presented are mean proportions ± standard errors. Data for separate cultivars were combined due to small sample sizes and proportions were analysed among sublocations on leaves. Different letters indicate a significant difference (*P* < 0.05), according to Fisher's protected least significant difference (LSD) test, in the proportion of eggs allocated to either leaf surface.

### 3.2 Larval performance

Of the 300 neonate larvae that were placed on plants, 247 (82%) were found after 72 h. Twenty‐three of the recovered larvae had died. From the 224 live larvae we recorded at the end of the experiment, 222 (99%) were located on or inside a floral structure. Of the remaining two larvae, one was located on a leaf and the other was found on the plant stem. Final larval location did not differ due to cultivar (*F*_2,20_ = 0.52, *P* = 0.60) or larval placement location (*F*_1,20_ = 1.82, *P* = 0.19).

**Figure 5:**
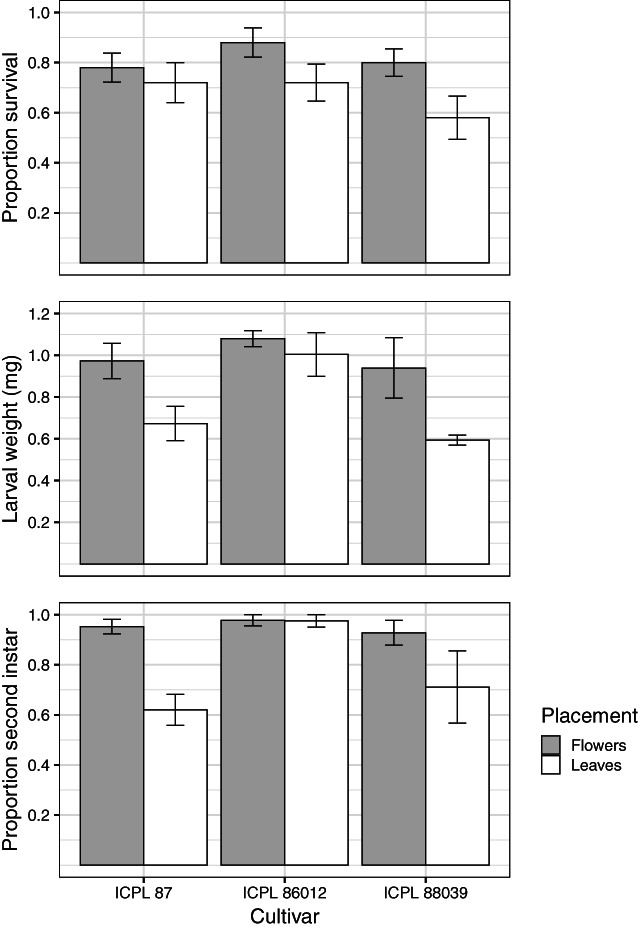
Larval performance measures from the neonate placement experiment, recorded at 72 h after placing neonates on pigeonpea plants. Grey bars indicate neonates placed on flowers, whereas white bars indicate larvae placed on leaves. Values shown are means and error bars represent standard errors. Detailed results are presented in the text, Table 5, and Tables S3 & S4.

Pigeonpea cultivar and larval placement location both affected the larval performance measures we recorded in the experiment (Table 5; Fig. 5). There was no difference in larval survival among pigeonpea cultivars (*F*_2,20_ = 2.03, *P* = 0.16). However, neonate placement location did influence larval survival, with more live larvae found on plants when neonates were placed on flowers (82%) compared to when larvae were placed on leaves (67%) (*F*_1,20_ = 10.80, *P* = 0.004; Fig. 5; Table S3).

**Table 5:** Analysis of variance (ANOVA) results for the neonate performance experiment showing the effect of pigeonpea cultivar and larval placement location on the final response variables measured at 72 h – final larval location (proportion in a floral structure), larval survival, larval weight, and larval development (proportion of larvae that reached second instar)

	df	Final larval location		Performance measure					
				Survival		Weight		Development	
		*F*	*P*	*F*	*P*	*F*	*P*	*F*	*P*
Cultivar	2	0.53	0.60	2.03	0.16	7.26	**0.004**	4.7	**0.021**
Placement treatment	1	1.82	0.19	10.80	**0.004**	14.86	**<0.001**	11.58	**0.003**
Cultivar × placement treatment	2	0.53	0.60	1.09	0.35	1.78	0.19	3.20	0.06
Block (replicate)	4	0.74	0.58	4.71	**0.008**	3.23	**0.03**	1.61	0.21

Values in bold indicate a significant difference (*p* < 0.05).

Larval weight was affected by both cultivar (*F*_2,20_ = 7.26, *P* = 0.004) and placement (*F*_1,20_ = 14.86, *P* < 0.001) treatments. Larval development was also affected by cultivar (*F*_2,20_ = 4.7, *P* = 0.021) and placement (*F*_1,20_ = 11.58, *P* = 0.003) treatments. Larvae weighed more and developed faster when placed on flowers compared to leaves (Table S3), and larvae developed faster and weighed more on ICPL 86012 compared with the other cultivars (Table S4). There were no significant cultivar × placement location interaction effects for any of the larval response variables (Table 5).

### 3.3 Quantifying trichomes

The types and densities of trichomes differed among the pigeonpea cultivars and plant structures (Table 6). Flower calyxes had all four types of trichomes, adaxial leaf surfaces had a high densities of Type C trichomes but Types A and B were also present, abaxial leaf surfaces had very high densities of Type C trichomes with Type B trichomes also detected. Trichomes were absent from flower petals. On flower calyxes the density of Type A (*F*_2,27_ = 24.04, *P* < 0.001), Type B (*F*_2,27_ = 9.98, *P* < 0.001), Type C (*F*_2,27_ = 3.55, *P* = 0.043), and Type D (*F*_2,27_ = 23.05, *P* < 0.001) trichomes all differed among cultivars. ICPL 87 calyxes had the most Type A trichomes, but the fewest of Types B, C, and D. On adaxial leaf surfaces the only difference detected was in the density of Type A trichomes (*F*_2,27_ = 5.12, *P* = 0.013), of which ICPL 86012 had the most. On abaxial leaf surfaces, cultivars varied in their density of Type B trichomes (*F*_2,27_ = 18.18, *P* < 0.001), of which ICPL 88039 had the highest density.

**Table 6:** Trichome measurements from different plant structures of three pigeonpea cultivars

Structure	Location	Cultivar	Trichome density (trichomes per mm^2^)			
			Type A (long glandular)	Type B (globular glandular)	Type C (short non‐glandular)	Type D (long non‐glandular)
Flower	Calyx	ICPL 87	2.09 ± 0.36 a	1.28 ± 0.27 b	145.49 ± 5.85 b	2.47 ± 0.51 b
		ICPL 86012	0.23 ± b	3.16 ± 0.20 a	174.72 ± 6.46 a	6.81 ± 0.54 a
		ICPL 88039	0.23 ± b	2.54 ± 0.41 a	164.27 ± 10.47 ab	6.78 ± 0.51 a
Leaf	Adaxial surface	ICPL 87	0.21 ± 0.09 ab	3.20 ± 0.29 a	231.68 ± 17.77 a	0
		ICPL 86012	0.49 ± 0.17 a	3.32 ± 0.42 a	232.75 ± 5.41 a	0
		ICPL 88039	0 b	4.31 ± 0.36 a	246.40 ± 12.25 a	0
	Abaxial surface	ICPL 87	0	11.14 ± 0.57 b	437.33 ± 13.4 a	0
		ICPL 86012	0	8.92 ± 0.59 b	452.48 ± 20.80 a	0
		ICPL 88039	0	16.17 ± 1.26 a	459.52 ± 26.08 a	0

Values presented are means followed by ± standard errors. Different letters indicate a significant (*P* < 0.05) difference, according to Fisher's protected least significant difference (LSD) test, in the density of a trichome type among cultivars for that plant structure and location (i.e. flower calyx, adaxial leaf surface, or abaxial leaf surface). Results of analysis of variance are presented in the text.

## 4 DISCUSSION

Female *H. armigera* moths did not exhibit a strong oviposition preference among the pigeonpea cultivars we examined. In the oviposition choice experiment slightly more eggs were laid on ICPL 88039, despite this cultivar having been reported as having moderate ovipositional antixenosis.^35^ On all cultivars moths laid most of their eggs on floral structures and larvae experienced greater survival, growth, and development when neonates were placed on flowers (simulating oviposition onto a flower) compared with when they were placed on leaves. Furthermore, larvae developed faster and grew to a larger size when feeding on cultivar ICPL 86012, despite this variety having been reported to have moderate resistance against *H. armigera*.^32^

In both oviposition choice and no‐choice experiments, *H. armigera* did not display ovipositional non‐preference against pigeonpea cultivars that have been reported to display antixenosis.^33^, ^35^ Sison *et al*.^33^ reported that four times the number of eggs were laid on ICPL 87 compared with ICPL 86012 in choice experiments and almost twice as many eggs were laid on ICPL 87 compared with ICPL 86012 in no‐choice tests. Similarly, Kumari *et al*.^35^ reported that ICPL 87 received significantly more eggs than ICPL 88039 under no‐choice and multichoice tests, but not paired tests. Our results did not replicate these findings.

In terms of the distribution of eggs among plant parts, moths in our study strongly targeted their eggs to floral structures, as has been shown previously for pigeonpea.^50^ The higher proportion of eggs laid on ICPL 88039 bud initials (Fig. 4) is likely due to the significantly larger number of bud initials present on ICPL 88039 plants (Table 2), indicating that plant architecture may influence moth oviposition behaviour. ICPL 88039 is an indeterminate cultivar of pigeonpea, whereas both ICPL 87 and ICPL 86012 are determinate. Indeterminate pigeonpea cultivars flower acropetally along a branch (or mainstem) and as a result, plants have many bud initials (Fig. 1(b)). Determinate varieties, however, flower basipetally and growing points turn into terminal racemes when plants commence flowering (Fig. 1(a)). The relationship between *H. armigera* oviposition and pigeonpea plant architecture has not been explored in detail and future research should examine how plant architecture may influence plant susceptibility to *H. armigera*.

Trichomes on pigeonpea plant structures may influence oviposition site selection by *H. armigera* moths,^50^ as *H. armigera* prefers to oviposit on ‘hairy’ surfaces.^8^ In our study, moths were recorded laying eggs on trichome dense sites (calyxes, leaf undersides) along with trichome‐absent sites (flower petals) (Tables 3 and 4). More eggs were laid on calyxes on bud initials and buds, but eggs were evenly distributed between petals and calyxes on flowers and spent flowers (Table 3). Due to our sample sizes, we were unable to compare the distribution of eggs at sublocations among cultivars, which is worthy of future investigation. Trichomes may be used by female moths to locate an appropriate plant structure (e.g. a flower) for oviposition. However, based on our data moths do not appear to discriminate where the eggs are laid within a floral structure (e.g. calyx or petal). This may be due to moth morphology, and the precise positioning of an egg might be constrained by accessibility of the moth's ovipositor. The available surface area of the different structures may also play a role. For instance, the surface area of petals on buds are small, but larger on flowers and spent flowers. Based on our results we cannot conclude the role trichomes play in oviposition site selection. Moths likely use a series of cues – volatiles and visual cues pre‐alighting, then tactile cues post‐alighting.

In our neonate larval performance experiment pigeonpea cultivar affected early instar larval performance. Larvae feeding on ICPL 86012 weighed more and developed faster, despite ICPL 86012 having been reported as resistant.^32^ Sison and Shanower^32^ reported that larvae fed on ICPL 86012 experienced lower larval weights, pupal weights, and slower larval development than larvae fed on ICPL 87. Similarly, for ICPL 88039, Kumari *et al*.^34^ reported slower development (relative to ICPL 87) when larvae were fed leaves. However, when fed on flowers and pods *H. armigera* larvae performed better on ICPL 88039 than on ICPL 87 in terms of larval weight and larval mortality at 10 days post‐eclosion, although no differences were detectable at pupation and moth emergence.^34^ Our data did not support the finding that ICPL 87 is the most susceptible pigeonpea variety of those tested.^31^, ^32^, ^34^ Differences obtained between our study and the previously published studies may be due to the different *H. armigera* populations, as previous studies used laboratory‐reared Indian *H. armigera* compared with the Australian population we used. More likely, the differences among results are due to our study using whole plants as opposed to detached plant parts in assays.

The overwhelming majority of larvae were located feeding at flowers by the end of our larval performance experiment. Larval survival was greater when neonates were placed on pigeonpea flowers compared with leaves (Fig. 5; Table 5). Greater mortality of larvae placed on leaves may be due to larvae silking off leaves as they are not preferred feeding sites,^43^ or mortality occurring during the longer process of a larvae searching for a suitable feeding site (i.e. a flower) while navigating sites dense with plant defences (e.g. trichomes on leaves and calyxes). However, due to our study design and difficulties measuring larval ‘drop off’ behaviour,^51^ the causal mechanism of larval mortality remains unknown. The relationship between oviposition sublocation and mortality of larvae (and eggs) may also be worth investigating. If eggs are evenly distributed between trichome‐present calyxes and trichome‐absent petals, is there any difference among egg or larval mortality for those sublocations? Similarly, given 86% of eggs laid on leaf undersides, do eggs and larvae survive better when eggs are laid on the abaxial surface?

Trichomes have been identified as a potential resistance mechanism against *H. armigera* in pigeonpea, with evidence that non‐glandular trichomes provide resistance against pest feeding in wild varieties of pigeonpea.^25^, ^30^, ^31^, ^52^ *Helicoverpa armigera* larvae may have to remove trichomes before they are able to establish a feeding site^53^ and such structural defences can be important in regulating early instar caterpillar survival.^54^ The cultivars and plant structures examined for trichomes in our study differed in trichome identity and density (Table 6). Firstly, there was a strong effect of location consistent among cultivars, which has been reported previously.^30^ Secondly, there was also a cultivar effect – both ICPL 86012 and ICPL 88039 had more non‐glandular trichomes on their flower calyxes in comparison with ICPL 87. Romeis *et al*.^30^ reported that ICPL 86012 pods also had higher levels of non‐glandular trichomes than ICPL 87. However, the density of non‐glandular trichomes recorded in our study were well under the density that non‐glandular trichomes are found on wild *Cajanus* species.^30^, ^31^

In our neonate performance experiment, larvae were allowed to select their own feeding site, as opposed the constraints typically placed on them in standard ‘Petri dish assays’ where larvae are confined to feeding on a particular plant part.^36^, ^37^, ^38^, ^39^, ^40^ Previous studies have shown that first instar *H. armigera* larvae have a behavioural tendency to move upwards on plants and locate flowers as feeding sites.^41^, ^55^, ^56^, ^57^ In our study, after 72 h, regardless of their initial placement location, almost all larvae were found feeding inside flowers, sites devoid of trichomes. Therefore, at flowering the potential of trichomes to regulate *H. armigera* larval establishment may be limited. However, as larvae develop, they ultimately switch from feeding on flowers to feeding on pods.^58^ Pigeonpea pods are densely covered with trichomes,^30^, ^52^ and consequently trichomes on pods may provide a more applicable resistance mechanism. Future experiments should examine when larvae commence pod‐feeding and the potential role of trichomes to inhibit pod‐feeding.

A large body of published research has examined host–plant resistance against *H. armigera* in pigeonpea.^25^ A Scopus search for ‘Helicoverpa+armigera+Cajanus+resistance’ identifies 33 relevant papers published in the last 10 years, with many more published previously and many published in non‐indexed journals. Sharma *et al*.^36^ is perhaps the seminal paper examining host–plant resistance against *H. armigera* in a range of crops using a laboratory assay – using plastic cups rather than Petri dishes. After obtaining drastically different results in laboratory bioassays and field plots, the authors state ‘the detached leaf assay did not seem to be a proper test to screen for resistance to *H. armigera* in pigeonpea.’ Yet numerous papers cite this reference when using a detached leaf bioassay for pigeonpea! Few of the published studies examining host–plant resistance in pigeonpea consider the behaviour of *H. armigera* and instead rely on detached plant part assays. The data presented in our study indicate that ovipositing *H. armigera* moths target most of their eggs to floral structures, sites where unrestricted larvae preferentially feed, and appear superior for larval performance. Future work aiming to identify host–plant resistance in pigeonpea against *H. armigera* should incorporate the behavioural preference of *H. armigera* moths and larvae into experimental evaluations.

## CONFLICT OF INTEREST

The authors declare no conflict of interest.

## Supporting information


**Figure S1:** Box‐and‐whisker plot displaying the number of *Helicoverpa armigera* eggs counted in the oviposition no‐choice experiment on three cultivars of pigeonpea. Bold horizontal bars represent the median, the grey box represents the interquartile range, and the points display values for individual replicates.
**Table S1:** Ingredients list for the artificial diet used for feeding the larvae of the *Helicoverpa armigera* culture
**Table S2**: Number of female *Helicoverpa armigera* moths that did and did not lay eggs in the oviposition no‐choice experiment
**Table S3:** Larval performance measures (survival, weight, development) 72 h after placement of neonates at different placement locations (leaves or flowers). Values represent means ± standard errors. Different letters indicate a significant (*P* < 0.05) difference within a column according to Fisher's protected least significant difference (LSD) test.
**Table S4:** Larval performance measures (survival, weight, development) 72 h after placement of neonates on different cultivars. Values represent means ± standard errors. Different letters indicate a significant (*P* < 0.05) difference within a column according to Fisher's protected least significant difference (LSD) test.

## Data Availability

The data that support the findings of this study are available from the corresponding author upon reasonable request.
