# Supplementary material for: Helicoverpa armigera preference and performance on three cultivars of short‐duration pigeonpea (Cajanus cajan): the importance of whole plant assays
Source: Pest Manag Sci. 2022 Oct 27;79(2):627–37. doi: 10.1002/ps.7230 (PMC10092315; doi:10.1002/ps.7230)
Supplement: Supplementary file 1 — Figure S1: Box‐and‐whisker plot displaying the number of Helicoverpa armigera eggs counted in the oviposition no‐choice experiment on three cultivars of pigeonpea. Bold horizontal bars represent the median, the grey box represents the interquartile range, and the points display values for individual replicates. Table S1: Ingredients list for the artificial diet used for feeding the larvae of the Helicoverpa armigera culture Table S2: Number of female Helicoverpa armigera moths that did and did not lay eggs in the oviposition no‐choice experiment Table S3: Larval performance measures (survival, weight, development) 72 h after placement of neonates at different placement locations (leaves or flowers). Values represent means ± standard errors. Different letters indicate a significant (P < 0.05) difference within a column according to Fisher's protected least significant difference (LSD) test. Table S4: Larval performance measures (survival, weight, development) 72 h after placement of neonates on different cultivars. Values represent means ± standard errors. Different letters indicate a significant (P < 0.05) difference within a column according to Fisher's protected least significant difference (LSD) test. [file PS-79-627-s001.docx]

**Supplementary material**

**Table S1:** Ingredients list for the artificial diet used for feeding the larvae of the *Helicoverpa armigera* culture

| **Ingredient** | **Amount** |
| --- | --- |
| Agar | 50g |
| Boiled distilled water | 3325mL |
| Soybean flour | 340g |
| Raw wheat germ | 240g |
| Brewer’s yeast | 200g |
| L-Ascorbic acid | 12g |
| Nipagin | 12g |
| Sorbic acid | 4g |
| Mould inhibitor  (Propionic acid. Phosphoric acid, Distilled water (7:1:9)) | 11mL |

**TABLE S2**: Number of female *H. armigera* moths that did and did not lay eggs in the oviposition no-choice experiment

| **Cultivar** | **Number of moths that laid eggs (% of total moths tested)** | **Number of moths that did not lay eggs** | **Total moths tested** |
| --- | --- | --- | --- |
| ICPL 87 | 14 (40%) | 21 | 35 |
| ICPL 86012 | 12 (35.3%) | 22 | 34 |
| ICPL 88039 | 8 (38.1%) | 13 | 21 |
| Total | 34 (37.8%) | 56 | 90 |

**TABLE S3:** Larval performance measures (survival, weight, development) 72h after placement of neonates at different placement locations (leaves or flowers). Values represent means ± standard errors. Different letters indicate a significant (p<0.05) difference within a column according to Fisher’s protected LSD test.

| **Placement location** | **Performance measure** | | |
| --- | --- | --- | --- |
|  | **Survival (proportion)** | **Weight (mg)** | **Development (proportion second instar)** |
| Leaves | 0.67±0.05 b | 0.76±0.06 b | 0.77±0.06 b |
| Flowers | 0.82±0.03 a | 1.00±0.06 a | 0.95±0.02 a |

**TABLE S4:** Larval performance measures (survival, weight, development) 72h after placement of neonates on different cultivars. Values represent means ± standard errors. Different letters indicate a significant (p<0.05) difference within a column according to Fisher’s protected LSD test.

| **Cultivar** | **Performance measure** | | |
| --- | --- | --- | --- |
|  | **Survival (proportion)** | **Weight (mg)** | **Development (proportion second instar)** |
| ICPL 87 | 0.75±0.05 a | 0.82±0.07 b | 0.79±0.06 b |
| ICPL 86012 | 0.80±0.05 a | 1.04±0.05 a | 0.98±0.02 a |
| ICPL 88039 | 0.69±0.06 a | 0.77±0.09 b | 0.82±0.08 b |


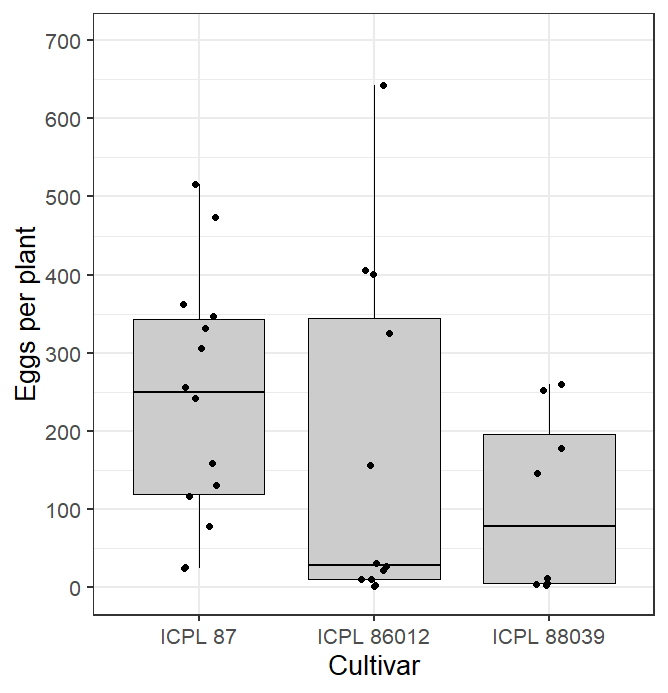


**FIGURE S1:** Box-and-whisker plot displaying the number of *H. armigera* eggs counted in the oviposition no-choice experiment on three cultivars of pigeonpea. Bold horizontal bars represent the median, the grey box represents the interquartile range, and the points display values for individual replicates.
